# A Stakeholder-Informed Approach to the Identification of Criteria for the Prioritization of Zoonoses in Canada

**DOI:** 10.1371/journal.pone.0029752

**Published:** 2012-01-06

**Authors:** Victoria Ng, Jan M. Sargeant

**Affiliations:** Centre for Public Health and Zoonoses, and Department of Population Medicine, Ontario Veterinary College, University of Guelph, Guelph, Canada; Monash University, Australia

## Abstract

**Background:**

Zoonotic diseases account for over 60% of all communicable diseases causing illness in humans and 75% of recently emerging infectious diseases. As limited resources are available for the control and prevention of zoonotic diseases, it is necessary to prioritize diseases in order to direct resources into those with the greatest needs. The selection of criteria for prioritization has traditionally been on the basis of expert opinion; however, details of the methods used to identify criteria from expert opinion often are not published and a full range of criteria may not be captured by expert opinion.

**Methodology/Principal Findings:**

This study used six focus groups to identify criteria for the prioritization of zoonotic diseases in Canada. Focus groups included people from the public, animal health professionals and human health professionals. A total of 59 criteria were identified for prioritizing zoonotic diseases. Human-related criteria accounted for the highest proportion of criteria identified (55%), followed by animal-related criteria (26%) then pathogen/disease-related criteria (19%).

Similarities and differences were observed in the identification and scoring of criteria for disease prioritization between groups; the public groups were strongly influenced by the individual-level of disease burden, the responsibility of the scientific community in disease prioritization and the experiences of recent events while the professional groups were influenced by the societal- and population-level of disease burden and political and public pressure.

**Conclusions/Significance:**

This was the first study to describe a mixed semi-quantitative and qualitative approach to deriving criteria for disease prioritization. This was also the first study to involve the opinion of the general public regarding disease prioritization. The number of criteria identified highlights the difficulty in prioritizing zoonotic diseases. The method presented in this paper has formulated a comprehensive list of criteria that can be used to inform future disease prioritization studies.

## Introduction

Zoonotic diseases are defined by the World Health Organization (WHO) as those that are naturally transmitted between vertebrate animals and humans. Zoonotic diseases account for over 60% of all communicable diseases causing illness in humans and 75% of recently emerging infectious diseases [1], [2]; each disease posing a varying degree of threat to public health. As limited resources are available for research, surveillance, control and prevention of zoonotic diseases, it is necessary to prioritize diseases in order to direct resources into those with the greatest needs.

A number of studies have attempted to methodically prioritize communicable diseases and pathogens of national and international public health concern 3,4,5,6,7,8,9,10. More recently, studies have focused on the prioritization of zoonotic diseases and pathogens [11], [12], [13], [14]. Although methodological approaches differ, priority-setting exercises typically follow a series of steps, these include (i) selecting a group of diseases/pathogens for prioritization; (ii) identifying a list of appropriate and measurable criteria to assess diseases/pathogens; (iii) defining a range of levels for each criterion; (iv) determining the relative importance by means of a weight or score for each level within each criterion; (v) assigning weights and/or scores on the selected criteria and levels and aggregating to produce an overall score for each disease/pathogen; and (vi) ranking diseases/pathogens by their overall score to derive a recommended list for prioritization. A cut-off score may apply for the inclusion or exclusion of diseases/pathogens from the priority list.

In a review of prioritization studies conducted between 1997 and 2011 (Tables 1 and 2), 11 studies identified a list of criteria to assess diseases/pathogens (step (ii)). The number of criteria ranged from 5 to 12 and was primarily selected on the basis of expert opinion; however, details of the methods used to identify those criteria from expert opinion were not published.

**Table 1:** Summary of methods used in criteria identification in prioritization exercises conducted between 1997 and 2004.

Country or region (Study year)	Diseases or pathogens of interest	Number of criteria identified	Methods used in criteria identification	Reference(s)
United Kingdom (1997)	33 communicable diseases and 8 generic disease groups	6	Not published, expert opinion (n = ?)	[5]
			“*The questionnaire's validity and its appropriateness as a tool was assessed by experts in communicable diseases to ensure that it covered the main areas and criteria needed for the priority setting exercise*.”	
United Kingdom (1999)	58 pathogens or communicable diseases and 11 generic disease groups	5	Not published, expert opinion (n = ?)	[4]
			“*The five criteria used in the survey to assess importance are similar to criteria that have been used in other priority setting exercises*.”	
Canada (2000)	43 communicable diseases	10	Not published, expert opinion and consensus of the subcommittee (n = ?).	[8]
			“*The subcommittee established 10 criteria to measure the importance of each disease*.”	
France (2000–2001)	37 non-food borne zoonoses	6	Not published, expert opinion (n = 10)	[11]
			“*An expert group decided on a set of scientific criteria that would present objective arguments to decision makers.*” [Translated from French]	
WHO Eastern Europe (7 countries) (2002)	53 communicable diseases	8	Not published, expert opinion (n = 24)	[3]
			“ *A total of eight criteria for assessment of importance were selected …*” [no mention of the criteria selection process but the study was conducted by a panel of experts]	
Germany (2004)	85 pathogens	12	Not published, expert opinion (n = 11)	[9], [15]
			“*As the main purpose of our work is to guide surveillance and research activities in the field of infectious-disease control and epidemiology in Germany, it is not surprising that most of our categories relate strongly to public health in general and to epidemiology in particular*.”	

**Table 2:** Summary of methods used in criteria identification in prioritization exercises conducted between 2005 and 2011.

Country or region (Study year)	Diseases or pathogens of interest	Number of criteria identified	Methods used in criteria identification	Reference(s)
Canada (2005)	48 communicable diseases	10	Not published, expert opinion and consensus of the subcommittee (n = ?) [8]	[7]
			Continuation of the work conducted in 2000 [8]	
France (2005–2008)	37 non-food borne zoonoses	6	Not published, expert opinion (n = 16)	[14]
			Continuation of the work from the initial meeting in 2000–2001 [11].	
Belgium (2008)	51 food- and water-borne zoonotic pathogens	5	Not published, expert opinion (n = ?)	[12]
			“*The choice of the criteria was made from the viewpoint to have a well-balanced representation of public and animal health criteria and to add a criterion “food” to comply with the aim of the study. Socioeconomic aspects in relation to public and animal health were also taken into consideration*.”	
The Netherlands (2010)	86 emerging zoonotic pathogens	7	Not published, expert opinion (n = ?).	[13]
			“*We quantified the risk to public health of emerging zoonoses by applying seven criteria that covered the complete pathway from introduction to societal impact.*”	
			“*The model for priority setting presented here is based on criteria reflecting the epidemiology and societal impact of zoonotic diseases. Risk perception by the general public is not included in this model, but may post additional challenges to policy makers. Further work to include risk perception as a second dimension in the priority model is recommended*.”	
Germany (2011)	127 pathogens	10	Not published, expert opinion (n = 11+72)	[10]
			“*The twelve criteria used during our previous prioritization process in 2004* [9], [15] *were further modified according to the feedback received from a broad group of different experts* [27]. *The newly suggested criteria and their three-tiered definitions were then reviewed by internal and external experts.*”	

As the success of a prioritization exercise is largely determined by having an appropriate list of criteria to assess diseases and pathogens, the methods for deriving the list of criteria should be transparent and scientifically driven. Although expert opinion was the preferred method in the selection of criteria for prioritization (Tables 1 and 2), many of these studies questioned whether all potential criteria were considered and whether the entire range of criteria can be captured impartially by expert opinion [4], [11], [12], [13], [15]. Expert groups acknowledge their priorities may not reflect the priorities concerning the general public or decision makers, particularly under social or political pressure [11], [12], [13]. It is therefore rational to include these stakeholders in developing a list of criteria for disease prioritization. Identifying an approach, that is transparent, reproducible and engages the collective opinion of content experts, decision makers and the general public, may lead to the selection of more appropriate criteria for a scientifically valid and robust prioritization exercise.

The first objective of this paper was to identify criteria for the prioritization of zoonotic diseases in Canada. Multiple focus groups comprising people from the public, animal health professionals and human health professionals were conducted to achieve this objective. The second objective was to explore the similarities and differences in responses between the public and the professional focus groups.

## Methods

Focus groups were used to identify a list of criteria to prioritize zoonoses [16]. Six focus groups were conducted over a five-week period in February and March of 2010, each group comprising eight to ten individuals (Table 3). A total of 54 individuals participated in the focus groups. Written informed consent was obtained from each participating individual. The groups were selected to reflect a range of demographic and professional characteristics. The number of groups were selected with the goal of reaching theoretical saturation [17], [18].

**Table 3:** Demographic characteristics of focus group participants.

Focus Group Number	Public or Professional	Number of individuals in the group	Males	Females	Average age (range)	Highest level of Education	Number of criteria identified as a group
1	Public	9	6	3	38.3 (21 to 56)	High school (4), college diploma (2), Bachelors degree (2), Masters degree (1); all in unrelated fields*	24
2	Public	9	5	4	41.4 (20 to 70)	High school (2), college diploma (3), Bachelors degree (4); all in unrelated fields*	27
3	Public	10	4	6	40.2 (21 to 67)	High school (5), college diploma (1), Bachelors degree (3), Masters degree (1); all in unrelated fields*	24
4	Professional (animal health experts and decision-makers)	10	8	2	47.7 (39 to 59)	PhD (7) and/or DVM/DVSc (7)	33
5	Professional (human health experts and decision-makers)	8	5	3	47.1 (33 to 52)	Diploma (1), Bachelors degree (1), Masters (1), PhD (4) and/or MD (1)	25
6	Professional (mix of animal and human health experts and decision-makers)	8 (four animal health and four human health professionals)	3	5	42.7 (30 to 58)	Masters (1), PhD (4) and/or MD (1) and/or DVM/DVSc (3)	31
**Total**	**-**	**54 (total); 9 (mean)**	**31 (57%)**	**23 (43%)**	**42.8 (mean)**	**-**	**164 (total); 27 (mean)**

*Excluded fields include medical and veterinary sciences, epidemiology and public health sciences, infectious diseases research including laboratory work on infectious diseases, nurses, and dentists.

Three groups comprised of individuals from the general public. These individuals were screened and disqualified if they were employed in any of the following fields: medical science, veterinary science, epidemiology and public health sciences, infectious diseases research including laboratory work on infectious diseases, nurses, dentists and animal health technicians. Two recruitment agencies were enlisted to recruit individuals by telephone in the three public focus groups (one group was conducted in Guelph, Canada while the remaining two were conducted in Toronto, Canada). Recruitment involved the random selection of individuals from an in-house database with specifications to include a range of demographic characteristics (gender, age and educational background) in each group. The response rate during the screening process was not requested or available for these focus groups. A financial incentive (C$50) was given to public group participants. The remaining three groups comprised of individuals employed exclusively in these professional fields; these included infectious disease epidemiologists, academic and practicing physicians and veterinarians, human and animal health laboratory microbiologists, pathologists and technicians, public health practitioners and policymakers at the local, provincial and national level with at least five years of work experience. Individuals in the professional groups were recruited by one of the authors (VN) by email invitation. Recruitment involved targeting selected individuals representing the range of professions listed above in the local areas (Toronto and Guelph) rather than targeting all relevant healthcare professionals in the local region. The response rate was 86% (31 of 36 individuals approached), of these, 28 (78% of the total individuals approached) were available at the set times and dates of the focus groups. The professional group individuals were divided into one group of animal health professionals, one group of human health professionals and the last group comprising a mix of the two. No financial incentive was given to professional group participants.

A total of 28 people participated in the public focus groups and 26 professionals participated in the professional focus groups (Table 3). There were slightly more males than females across all groups (57% to 43%) and within public groups (54% to 46%) and professional groups (62% to 38%). The age range was 20 to 70 years in the public groups and 30 to 59 years in the professional groups. The professional groups encompassed a narrower age range because only individuals in active employment with at least 5 years of work experience in their profession were selected to participate. The public groups were more likely to be high school and college graduates or hold a Bachelor's degree in an unrelated field (these fields were medical and veterinary sciences, epidemiology and public health sciences, infectious diseases research including laboratory work on infectious diseases, nursing and dentistry) while the professional groups were more likely to hold a PhD in the related fields and/or a professional degree (DVM, DVSc or MD).

An experienced moderator conducted all six focus groups using a prepared script (available as Information S1) to ensure consistency between groups. The group discussions were audio taped and transcribed for validation of the group exercises. A nominal group technique [19] was used to structure the group discussion. Participants were presented with the research question - ‘*What are important characteristics of zoonotic diseases that should be considered in disease prioritization*?’ and were informed that the objective of the session was to formulate a list of criteria, in order of importance, that could be used to prioritize zoonotic diseases for their control and prevention in Canada. The exact mode of control and prevention was not specified but could include regulation, management, vaccination, laboratory diagnosis, research and surveillance. A zoonosis was defined as a disease that is naturally transmitted between humans and animals, including vector-borne and enteric diseases, as well as diseases of animal-origin but primarily circulating amongst humans (for example, SARS and H1N1).

The first half of the focus group session required participants to identify a list of criteria to prioritize zoonoses; participants were presented with a comparison of two zoonoses (H1N1 and H5N1) as a prompt for identifying a list of criteria. Group discussion at this stage was discouraged and participants were asked to identify a list of criteria without consultation from other group members. Participants were given 20 minutes for this step. Once completed, participants were invited to share one criterion from their list in a round-robin format until every criterion on each participant's list had been shared. To avoid duplication, participants were asked to only share one criterion that the group had not previously shared. A flip chart was used to document the group's combined list of criteria. On completion, participants were given the opportunity to explain why certain criteria were identified and a group discussion was encouraged to clarify and discuss the list. The group was then asked to review the list and to remove or merge criteria that appeared to overlap.

The second half of the focus group session was used to apply scores to the list of criteria identified by the group. Participants were asked to score on a scale of 1 (least important) to 9 (most important) each criterion identified. As with the formulation of the list of criteria, participants were asked to score each criterion on their own. Scores from each participant were tabulated and the mean score per criterion was used to create a ranked list of criteria; this list was presented back to the group for discussion. Participants were encouraged to discuss why some criteria ranked low while others ranked high. For the three public focus groups, a second round of scoring was undertaken giving participants the opportunity to re-rank each criterion in light of group discussion. Due to time constraint, the three professional groups only participated in one round of scoring.

The two authors (VN and JMS) independently merged and condensed the lists of criteria from each focus group into one overall list by combining similar criteria together; this process was informed by reviewing the transcripts from the focus group discussions. The decisive factors for combining similar criteria together were that they described the same characteristic (for example, “ability or potential to control the disease” and “efficacy of current control methods”) and/or that they overlapped in their definitions (for example, “case-fatality rate in humans” and “mortality caused by the disease in people” were merged into the criterion *mortality characteristics in humans*). Although case-fatality and mortality have distinct epidemiological definitions, both include the total number of deaths caused by the disease in their derivation, which would result in duplicating the characteristic ‘total number of deaths caused by the disease’ in the final list of criteria had both been included.

On completion, the two authors examined their condensed lists together and reached consensus on the number of unique criteria identified across the six focus groups. A score for each unique criterion was calculated by averaging the mean scores across duplicate criteria within each unique criterion. The authors also divided the unique criteria into nine themes for analysis by consensus. These themes were the burden of illness, disease epidemiology, control measures, socioeconomic impact, diagnosis, disease knowledge, public awareness and concerns, international considerations and local considerations. Although the burden of illness can be considered a theme within disease epidemiology, it encompasses the range of individual-level physical burden of the disease; these include mortality, severity, and the duration of illness. Disease epidemiology was considered to be aspects of the force of infection driving disease burden at the population-level; these include incidence, prevalence, transmission potential, speed of transmission within individuals, speed of transmission between individuals, immunogenicity, endemicity and high-risk groups.

## Results

Six separate lists of criteria were obtained from the focus groups; each group identified between 24 and 33 criteria during the exercise, a total of 164 criteria were identified across all groups (Table 3). As expected, criteria duplication was observed across groups. A final list of 59 unique criteria for prioritizing zoonotic diseases elicited from six focus groups using the nominal group technique [19] are presented in Tables 4, 5 and 6. The criteria are presented by the nine themes that are further divided into human-related, animal-related or pathogen/disease-related categories. Criteria are presented in order of highest mean score by theme, then by human-, animal- or pathogen/disease-related categories. The theme with the most number of criteria identified was disease epidemiology (43%), followed by control measures (17%), socioeconomic impact (12%), disease burden (10%), and diagnosis (7%). Human-related criteria accounted for the highest proportion of criteria identified (55%), followed by animal-related criteria (26%) then pathogen and disease-related criteria (19%).

**Table 4:** Fifty-nine unique criteria identified collectively by six focus groups for prioritizing zoonotic diseases in Canada (Part 1).

	Criteria	Theme	Human, animal, pathogen or disease	Public Groups			Professional Groups			Mean Score∧	Frequency#
				1	2	3	AH+	HH+	A/H+		
1	Mortality characteristics in humans	Burden of illness	Human	2‡	1	1		1		8.6	4
2	Severity of illness in humans	Burden of illness	Human	1	1	1	1	1	1	7.6	6
3	Duration of illness in humans	Burden of illness	Human		2					6.0	1
4	Co-infection in humans	Burden of illness	Human	1						5.1	1
5	Reproductive consequences in humans	Burden of illness	Human			1				4.4	1
6	Severity of illness in animals	Burden of illness	Animal			1	1	1	1	6.8	4
7	Combination of disease risk and probability of infection	Disease epidemiology	Human				1			8.6	1
8	Potential for animal-to-human transmission	Disease epidemiology	Human				1		1	8.2	2
9	Speed of transmission between humans	Disease epidemiology	Human	1	1	1				8.0	3
10	Incidence or prevalence in humans	Disease epidemiology	Human					1	1	7.5	2
11	Potential for human-to-human transmission	Disease epidemiology	Human			1	1	1	1	7.9	4
12	Risk of transmission in humans	Disease epidemiology	Human					1		7.6	1
13	Speed of disease spread in individuals (incubation period)	Disease epidemiology	Human	1	2	2				7.0	3
14	High-risk groups in humans	Disease epidemiology	Human	1	1	1	1	1	1	6.5	6
15	Immunogenicity in humans	Disease epidemiology	Human	1	1				1	5.0	3
16	Risk of endemicity in humans	Disease epidemiology	Human					1		5.0	1
17	Impact of climate change on vectors and animal hosts	Disease epidemiology	Animal				2			7.0	1
18	Incidence or prevalence in animals	Disease epidemiology	Animal					1	1	5.5	2
19	Specific types of animals involved	Disease epidemiology	Animal			1	1			6.3	2
20	Size of the reservoir host (animal and environment)	Disease epidemiology	Animal			1	2		2	6.2	3
21	Potential for animal-to-animal transmission	Disease epidemiology	Animal				1		2	6.1	2
22	Potential for human-to-animal transmission	Disease epidemiology	Animal				1	1	1	5.5	3
23	Risk of endemicity in animals	Disease epidemiology	Animal					1		5.1	1
24	High-risk groups in animals	Disease epidemiology	Animal				1			4.5	1

+ AH = animal health professional group, HH = human health professional group and A/H = mixed animal and human health professional group.

∧ Mean score on a scale of 1 being the least important to 9 being the most important.

# The number of focus groups that identified the unique criterion; 1 indicates only one focus group listed the criterion while 6 indicates all focus groups listed the criterion.

‡ The number of times the unique criterion was identified by each focus group; 2 or more indicates the criterion was listed more than once but expressed in different terms by the focus group.

**Table 5:** Fifty-nine unique criteria identified collectively by six focus groups for prioritizing zoonotic diseases in Canada (Part 2).

	Criteria	Theme	Human, animal, pathogen or disease	Public Groups			Professional Groups			Mean Score∧	Frequency#
				1	2	3	AH+	HH+	A/H+		
25	Mode of transmission	Disease epidemiology	Pathogen/disease	1‡	1	1	1	1	1	7.2	6
26	Pathogenicity and virulence of the pathogen	Disease epidemiology	Pathogen/disease				1		2	6.7	2
27	Range of pathogens	Disease epidemiology	Pathogen/disease				1			6.8	1
28	Disease trend	Disease epidemiology	Pathogen/disease	1			1	1	1	6.2	4
29	Ability of the pathogen to mutate and adapt to change	Disease epidemiology	Pathogen/disease	1		1		2	3	5.8	4
30	Endemicity of the disease due to climate	Disease epidemiology	Pathogen/disease	1		1				4.1	2
31	Seasonality of the disease	Disease epidemiology	Pathogen/disease	1	1			1		3.5	3
32	Treatment and prevention in humans	Control measures	Human	2	4	2	2	2	1	7.2	6
33	Potential to eradicate the disease in humans	Control measures	Human		1	1			1	6.5	3
34	Visual cues to avoid the disease in humans	Control measures	Human	1	1	1				6.0	3
35	Surveillance in humans	Control measures	Human				1			5.9	1
36	Human cause versus a natural cause	Control measures	Human		1					5.9	1
37	Disease in human beyond control measures	Control measures	Human		1		1			5.8	2
38	Vaccine/antiviral manufacturing time	Control measures	Human	1						5.8	1
39	Potential to eradicate the disease in animals	Control measures	Animal			1				6.5	1
40	Treatment and prevention in animals	Control measures	Animal		1		2		1	6.2	3
41	Surveillance in animals	Control measures	Animal				1			5.6	1
42	Risk of bioterrorism	Socioeconomic impact	Human	1				1		6.7	2
43	Political impact of the disease in humans	Socioeconomic impact	Human					1		6.4	1
44	Risk to the food and water supply	Socioeconomic impact	Human	1	2					6.4	2
45	Socioeconomic burden of the disease in humans	Socioeconomic impact	Human	1	2	2	2	1	1	5.6	6
46	Psychological impact in humans	Socioeconomic impact	Human					1		5.4	1
47	Socioeconomic burden of the disease at the individual/farm level	Socioeconomic impact	Animal				1		1	5.8	2
48	Socioeconomic burden of the disease on the industry	Socioeconomic impact	Animal	1			1	1	2	6.3	4

+ AH = animal health professional group, HH = human health professional group and A/H = mixed animal and human health professional group.

∧ Mean score on a scale of 1 being the least important to 9 being the most important.

# The number of focus groups that identified the unique criterion; 1 indicates only one focus group listed the criterion while 6 indicates all focus groups listed the criterion.

‡ The number of times the unique criterion was identified by each focus group; 2 or more indicates the criterion was listed more than once but expressed in different terms by the focus group.

**Table 6:** Fifty-nine unique criteria identified collectively by six focus groups for prioritizing zoonotic diseases in Canada (Part 3).

	Criteria	Theme	Human, animal, pathogen or disease	Public Groups			Professional Groups			Mean Score∧	Frequency#
				1	2	3	AH+	HH+	A/H+		
49	Ease of diagnosis and lead-time in diagnosis in humans	Diagnosis	Human		1‡	1		1		6.0	3
50	Availability of diagnostic tests in humans	Diagnosis	Human				1			6.3	1
51	Test characteristics in humans (accuracy, multiple tests)	Diagnosis	Human						1	3.9	1
52	Availability of diagnostic tests in animals	Diagnosis	Animal				1			6.0	1
53	Scientific knowledge of the disease	Disease knowledge	Pathogen/disease	1	1			1	1	6.3	4
54	Public disruption	Public awareness and concerns	Human						1	7.7	1
55	Public awareness	Public awareness and concerns	Human	1						7.5	1
56	Public perception	Public awareness and concerns	Human				1	1	1	5.6	3
57	Geographic distribution of the disease	International considerations	Pathogen/disease	1	1	1		1		4.6	4
58	Geographic source of the disease	International considerations	Pathogen/disease			1				5.1	1
59	Disease occurring in jurisdiction of interest	Local considerations	Pathogen/disease				1			7.0	1
**TOTAL**				**24**	**27**	**24**	**33**	**25**	**31**	**6.2**	**141**
**Mean Score** ∧ **by groups (range)**				**6.2 (3.6 to 8.6)**			**6.2 (3.3 to 8.7)**			**-**	**-**

+ AH = animal health professional group, HH = human health professional group and A/H = mixed animal and human health professional group.

∧ Mean score on a scale of 1 being the least important to 9 being the most important.

# The number of focus groups that identified the unique criterion; 1 indicates only one focus group listed the criterion while 6 indicates all focus groups listed the criterion.

‡ The number of times the unique criterion was identified by each focus group; 2 or more indicates the criterion was listed more than once but expressed in different terms by the focus group.

The public groups identified fewer criteria (35) than the professional groups (46); of these, 22 were common, 13 were unique to the public groups and 24 were unique to the professional groups. Criteria relating to disease epidemiology, socioeconomic impact, diagnosis, public awareness and concerns and local considerations were identified more often in the professional groups (Tables 4, 5 and 6). Control measures, burden of illness, international considerations and disease knowledge were identified more often in the public groups. Human-related criteria accounted for the highest proportion of listed criteria in both the public and professional groups. The professional groups identified more animal-related criteria than the public groups. Due to the qualitative approach, the terms used to describe differences between groups, for example, more/less, higher/lower, are only numeric as no statistical comparisons were performed.

A mean score was calculated for each criterion; a score closer to 9 indicated the criterion was most important while a score closer to 1 indicated the criterion was least important. The mean score for all criteria across all groups was 6.2 ranging from 3.5 (*seasonality of the disease*) to 8.6 (*mortality characteristics in humans* and *combination of disease risk and probability of infection*) (Tables 4, 5 and 6). The mean score for the public groups was 6.2 ranging from 3.6 to 8.6. The mean score for the professional groups was also 6.2 ranging from 3.3 to 8.7.

The top ten criteria ranked by mean score by public and professional groups are presented in Table 7. *Mortality characteristics in humans* was the highest scored criteria in both the public and professional groups. Other shared criteria in the top ten between the two groups included *mode of transmission* and *potential for human-to-human transmission*. The bottom ten criteria ranked by mean score by public and professional groups are presented in Table 8. *Seasonality of the disease* was the lowest scored criteria in both the public and the professional groups. Other shared criteria in the bottom ten between the two groups included *geographic distribution of the disease* and *immunogenicity in humans*.

**Table 7:** Top 10 criteria rank-ordered by mean score by the public and professional focus groups.

Rank	Focus group	Criteria	Themes	Human, animal or pathogen/disease	Mean Score
1	Public	Mortality characteristics in humans+	Burden of illness	Human	8.6
2	Public	Speed of transmission between humans	Disease epidemiology	Human	8.0
3	Public	Treatment and prevention in humans	Control measures	Human	7.5
4	Public	Severity of illness in animals	Burden of illness	Animal	7.5
5	Public	Public awareness	Public concerns	Human	7.5
6	Public	Scientific knowledge of the disease	Disease knowledge	Pathogen/disease	7.5
7	Public	Mode of transmission+	Disease epidemiology	Pathogen	7.4
8	Public	Potential to eradicate the disease in humans	Control measures	Human	7.3
9	Public	Speed of disease spread in individuals (incubation period)	Disease epidemiology	Human	7.0
10	Public	Potential for human-to-human transmission+	Disease epidemiology	Human	6.9
1	Professional	Mortality characteristics in humans+	Burden of illness	Human	8.7
2	Professional	Combination of disease risk and probability of infection	Disease epidemiology	Human	8.6
3	Professional	Severity of illness in humans	Burden of illness	Human	8.4
4	Professional	Potential for human-to-human transmission+	Disease epidemiology	Human	8.2
5	Professional	Potential for animal-to-human transmission	Disease epidemiology	Human	8.2
6	Professional	Public disruption	Public concerns	Human	7.7
7	Professional	Risk of transmission in humans	Epidemiology	Human	7.6
8	Professional	Incidence or prevalence in humans	Disease epidemiology	Human	7.5
9	Professional	Socioeconomic burden of the disease in humans	Socioeconomic impact	Human	7.1
= 10	Professional	Impact of climate change on vectors and animal hosts	Disease epidemiology	Animal	7.0
= 10	Professional	Mode of transmission+	Disease epidemiology	Pathogen	7.0

+ Shared criterion identified as one of top ten criteria by both the public and the professional groups.

**Table 8:** Bottom 10 criteria rank-ordered by mean score by the public and professional focus groups.

Rank	Focus group	Criteria	Themes	Human, animal or pathogen/disease	Mean Score
35	Public	Seasonality of the disease+	Disease epidemiology	Disease	3.6
34	Public	Endemicity of the disease due to climate	Disease epidemiology	Disease	4.1
33	Public	Socioeconomic burden of the disease in humans	Socioeconomic impact	Human	4.1
32	Public	Reproductive consequences in humans	Burden of illness	Human	4.4
31	Public	Geographic distribution of the disease+	International considerations	Pathogen/disease	4.8
30	Public	Disease in human beyond control measures	Control measures	Human	4.8
29	Public	Disease trend	Disease epidemiology	Disease	5.0
28	Public	Co-infection in humans	Burden of illness	Human	5.1
27	Public	Geographic source of the disease	International considerations	Pathogen/disease	5.1
26	Public	Immunogenicity in humans+	Disease epidemiology	Human	5.2
46	Professional	Seasonality of the disease+	Disease epidemiology	Disease	3.3
45	Professional	Test characteristics in humans (accuracy, multiple tests)	Diagnosis	Human	3.9
44	Professional	Geographic distribution of the disease+	International considerations	Pathogen/disease	4.0
43	Professional	High-risk groups in animals	Disease epidemiology	Animal	4.5
42	Professional	Immunogenicity in humans+	Disease epidemiology	Human	4.6
41	Professional	Ease of diagnosis and lead-time in diagnosis in humans	Diagnosis	Human	4.9
40	Professional	Risk of endemicity in humans	Disease epidemiology	Human	5.0
39	Professional	Scientific knowledge of the disease	Disease knowledge	Pathogen/disease	5.1
38	Professional	Potential to eradicate the disease in humans	Control measures	Human	5.1
37	Professional	Risk of endemicity in animals	Disease epidemiology	Animal	5.1

+ Shared criterion identified as one of bottom ten criteria by both the public and the professional groups.

A list of criteria most frequently identified by all groups is presented in Table 9. Five criteria were identified by all six focus groups - *severity of illness in humans*, *treatment and prevention in humans*, *high-risk groups in humans*, *socioeconomic burden of disease in humans* and *mode of transmission*. For the public group, the criteria *speed of disease spread in individuals (incubation period)*, *speed of transmission between humans* and *visual cues to avoid the disease in humans* were identified by all three public groups but not in any professional groups. Conversely, the criteria *potential for human to animal transmission* and *public perception* were identified by all three professional groups but not in any public groups.

**Table 9:** List of criteria most frequently identified by the public and professional focus groups.

Focus group	Criteria	Themes	Human, animal or pathogen/disease	Frequency
Public	Severity of illness in humans+	Burden of illness	Human	3
Public	Treatment and prevention in humans+	Control measures	Human	3
Public	High-risk groups in humans+	Disease epidemiology	Human	3
Public	Socioeconomic burden of the disease in humans+	Socioeconomic impact	Human	3
Public	Mode of transmission+	Disease epidemiology	Pathogen	3
Public	Mortality characteristics in humans	Burden of illness	Human	3
Public	Geographic distribution of the disease	International considerations	Pathogen/disease	3
Public	Speed of disease spread in individuals (incubation period)*	Disease epidemiology	Human	3
Public	Speed of transmission between humans*	Disease epidemiology	Human	3
Public	Visual cues to avoid the disease in humans*	Control measures	Human	3
Professional	Severity of illness in humans+	Burden of illness	Human	3
Professional	Treatment and prevention in humans+	Control measures	Human	3
Professional	High-risk groups in humans+	Disease epidemiology	Human	3
Professional	Socioeconomic burden of the disease in humans+	Socioeconomic impact	Human	3
Professional	Mode of transmission+	Disease epidemiology	Pathogen	3
Professional	Severity of illness in animals	Burden of illness	Animal	3
Professional	Disease trend	Disease epidemiology	Disease	3
Professional	Potential for human-to-human transmission	Disease epidemiology	Human	3
Professional	Socioeconomic burden of the disease on the industry	Socioeconomic impact	Animal	3
Professional	Public perception*	Public concerns	Human	3
Professional	Potential for human-to-animal transmission*	Disease epidemiology	Animal	3

+ Shared criterion identified by all six focus groups.

*Unique criterion identified only by either the public or professional groups.

Criteria are not listed in any particular order. All listed criteria were identified by all three public or professional focus groups.

## Discussion

We present on a stakeholder-informed, mixed semi-quantitative and qualitative approach to identify a list of criteria that can be used to prioritize zoonotic diseases in Canada. A total of 59 unique criteria were identified in this exercise across six focus groups. Comparing the list of 59 unique criteria with the criteria used in previous prioritization studies (Tables 1 and 2), a much larger number of criteria were identified in this study than have been previously used, although many of these studies involved diseases exclusively in humans hence animal-related criteria would not be expected to be included in these studies. Further, previously published works focused on specific aspects of disease prioritization or a specialized group of diseases, for example prioritizing solely for surveillance [7], [8] or prioritizing emerging diseases only [13]. Finally, two or more criteria from previous studies could be combined into one of our unique criterion, for example, the severity of illness and duration of illness in humans were often combined into morbidity in humans while disease incidence could be combined with severity and mortality [3], [4], [5], [11], [12], [13], [14], thus reducing the number of criteria considered.

Similarities and differences were observed between the public and the professional focus groups. Amongst the similarities were the number of criteria identified within groups, the mean score across all identified criteria and the range in scores across all identified criteria (Tables 4, 5 and 6). The scores given to the most important criteria and least important criteria by groups were also near identical in score and range (Tables 7 and 8). The concordance in the mean score and range across the most salient and least salient criteria by groups indicate an overall agreement with the use of the scoring system and the nominal group technique for the rating of criteria importance.

Similarities were also observed in the overlap of three identified criteria each in the top ten and bottom ten lists suggesting some agreement between the public and professional groups in the criteria that can inform disease prioritization. The agreement on the high scoring of *mortality characteristics in humans*, *mode of transmission* and *potential for human-to-human transmission* suggest these criteria are of greatest importance for both public and professional stakeholders. This was substantiated by the focus group discussions in which the implications for human mortality and the transmissibility of the disease in humans dominated the discussion on what should be considered the most influential characteristics for disease prioritization. Conversely, low scoring criteria - *seasonality of the disease*, *geographic distribution of the disease* and *immunogenicity in humans* did not dominate in the focus group discussions, this was not because these criteria were unimportant (in fact, one could argue that all 59 unique criterion identified in this study were important given their identification in the focus groups), but rather, in comparison to all other identified competing criteria, these criteria were deemed less important by both public and professional stakeholders. The arguments for reduced importance varied and included that the cooler climate in Canada was unlikely to support disease persistence throughout the year (*seasonality of the disease*), that diseases do not respect political boundaries and with global trade and travel, a disease in one country can spread across the world within days (*geographic distribution of the disease*), and that immunity status can be modified by vaccination or is only relevant for diseases where reoccurrence is of high concern (*immunogenicity in humans*).

More differences were observed than similarities between the public and the professional groups. Although the number of criteria identified within each group was similar, the professional groups collectively identified more criteria with over half of these unique to the group (Tables 4, 5 and 6). Further, the professional groups described criteria definitions in more detail than the public group. For example, for the criterion *socioeconomic burden of the disease in humans*, the definition for this criterion in the professional groups encompassed a range of direct and indirect costs to the burden of illness including “*the societal impact, the commerce impact, the psychological impact beyond the impact of the illness*”, “*cost of treating and managing people*”, “*impact on daily living*” and “*it's broader, indirect things, isolation, psychological impact, lost labour … it encompasses things that are more than just the direct cost of illness*”. In comparison, the public group's definition of socioeconomic burden was primarily the “*direct cost of treatment*” (identified by two groups), “*cost of prevention*” and the “*cost to control disease spread*”, although one group expanded the definition further to include the “*effect on the health care system including cost, hospital bed and logistics*”. The larger number of unique criteria identified and the broader criteria definition in the professional groups was not surprising due to the educational background and professional experiences relating to the prioritization of diseases in this group. The differences in criterion definition therefore influenced its scoring with the professional groups scoring *socioeconomic burden of the disease in humans* high (Table 7); largely on the basis of the impact of this criterion at the societal- and population-level while the public group scored this criterion low (Table 8); primarily on the basis of the impact of this criterion at the individual-level.

Differences could also arise from emphasis placed on who was responsible for addressing the criterion. For example, the criterion *scientific knowledge of the disease* scored high in the public groups (Table 7) but low in the professional groups (Table 8). Although the reasoning given when identifying the criterion were similar between the public (“*if it is a brand new disease and nobody knows anything about it, say in the 1980s and AIDS popped up and nobody knew anything about it, then it's a high priority for me*”) and the professionals (“*there is a need for research … how much is known generally, for example, if it is a brand new disease, like SARS was when it first came out, then I think it's going to be high priority*”), the scoring differed between the public and professional groups because the public group placed more emphasis on the responsibility of scientists in addressing the scientific knowledge of the disease. Some of the comments from the public groups included “*Scientists are the ones that are going to fix this problem if they came up with a cure*”, “*Because they* [scientists] *are the ones that are doing the research*” and “*If nothing is known, then it is a high priority for scientists in terms of finding funding for prioritization*”. In comparison, the professional groups made comments relating only to the criterion rather than who was responsible for addressing the criterion, in fact, responsibility was assumed for this group - “*if we know a lot versus if we don't know much, then maybe we need to prioritize it to know more*” and “*from a research point of view, how much do you need to know about a disease, so in other words, how much money needs to be spent on research*”.

The focus groups were conducted less than one year after the initial outbreak of the pandemic influenza H1N1 virus in April 2009 [20] and the subsequent rapid spread across the globe [21], [22]. The awareness of the rapid spread of the H1N1 pandemic was evident in two criteria identified in the public groups – *speed of disease spread in individuals (incubation period)* and *speed of transmission between humans*, these were also criteria that were only identified by all three public groups and not in any professional groups. In reference to the speed of transmission, one participant noted ‘*with speed of transmission I mean it originates from Central America and it's already in North America a week later*’. Direct reference to H1N1 was also made for the criteria *ability to mutate* – ‘*I was just thinking if the swine flu kind of mutated* [then it would be a problem].’ The criteria *how quickly can the vaccine or antiviral be manufactured*, although not made in direct reference to H1N1, likely reflected on the vaccine shortages throughout Canada during the H1N1 pandemic [23]. It was evident that the criteria identified by the public groups were influenced by recent events while this was not as apparent for the professional groups.

While recent events may influence the public groups, the professional groups were strongly influenced by public perception. The criterion *public perception* was identified by all three professional groups but not in any public groups. Although public perception was a central theme for the professional group discussions, it was often argued that public perception should not be a factor in disease prioritization, thus a low score. However, the professional group acknowledged that disease prioritization in practice was often driven by public and political pressure. This is best summed up by one of the professional participants: “*I think generally, politically, that is what happens* [priority is given to diseases of media attention]. *A disease may not be that important really in terms of morbidity and mortality, but if the public is panicking, then the government will do something*”.

There are limitations relating to the method described in this paper, first and foremost, there may have been a selection bias relating to how individuals were chosen to participate in the study; the use of an in-house recruitment agency database to recruit individuals from the public and targeting selected professionals in the local region rather than randomly selecting individuals from the general public and targeting all relevant healthcare professionals across Canada. Our study therefore makes the assumption that the local public and professional groups selected to participate are in fact representative of the Canadian public and Canadian healthcare professionals. There may have also been a response bias relating to those who agreed to participate in the study. The effect of these methodological biases on the study results is unknown. Second, the professional groups undertook one round of scoring while the public groups were asked to re-score their criteria after a group discussion on the first round of scoring. While this may influence the final scores within groups, the concordance in the mean score and range between the public and the professional groups indicate an overall agreement with the use of the scoring system regardless of the number of rounds of scoring that was undertaken. Additionally, the professional groups were asked whether their scores would change after their group discussion and it was unanimously agreed upon that their scores would not change. Conversely, the public groups all agreed on the value of having a group discussion to come up with a final round of scoring, the latter of which did change from the initial round of scoring.

The advantages and disadvantages of using the nominal group technique in focus groups have already been described by other papers [16], [19]. The major strengths in the nominal group technique are the ability to clarify and minimize the differences of opinion between multiple participants and ensuring equal participation in all members. The main weaknesses are that a certain amount of agreement must already exist in participating members and the amount of time necessary to prepare and execute the technique in each focus group. The method described in this paper has been used to develop criteria for consideration in other health care studies [24], [25], [26].

We presented on the use of focus groups to derive a list of criteria for the prioritization of zoonotic diseases in Canada. This was the first study to describe a semi-quantitative and qualitative mixed approach to deriving criteria for disease prioritization. This study was also the first to collectively engage public and professional stakeholders in disease prioritization. Similarities and differences were observed in the identification and scoring of criteria for disease prioritization between groups; the public groups were strongly influenced by the individual-level of disease burden, the responsibility of the scientific community in disease prioritization and the experiences of recent events while the professional groups were influenced by the societal- and population-level of disease burden and political and public pressure.

The number of unique criteria identified in this study highlights the number of factors that need to be considered jointly, and thus, the difficulty in prioritizing zoonotic diseases. The study showed that relying on expert opinion alone may limit the range of suitable criteria considered for disease prioritization. The method presented in this paper has formulated a comprehensive list of criteria that can be used to inform other disease prioritization studies.

## Supporting Information

Information S1 **Focus group script.**
(DOCX)
