# Supplementary material for: A Stakeholder-Informed Approach to the Identification of Criteria for the Prioritization of Zoonoses in Canada
Source: PLoS One. 2012 Jan 6;7(1):e29752. doi: 10.1371/journal.pone.0029752 (PMC3253104; doi:10.1371/journal.pone.0029752)
Supplement: Information S1 — Focus group script. (DOCX) [file pone.0029752.s001.docx]

**Supporting Information S1**

**Focus group script**

The following is written up on a board to be referred to during the focus group session:

**Research Question:** What are important characteristics of a zoonotic disease that should be considered in disease prioritization?

**Goal:** Formulate a list, in order of importance, of characteristics that can be used to prioritize zoonotic diseases against each other

**1. Welcome and Introduction (10 minutes)**

**Welcome and introduction** – *“Hi, my name is VN and I will be facilitating today’s focus group session. You have been invited to participate in this focus group session because [I am particularly interested in the opinion of people from the public about this research topic / of your academic background or professional role]. What I anticipate I will get out of this session will be valuable for informing my study survey instrument so your presence here today is greatly appreciated.”*

**Running Time** – *“The focus group will last for approximately one and a half hours.”*

**Study Background and Focus Group goal** – *“I am currently running a study on the prioritization of zoonotic diseases (explain the definition of a zoonotic disease for this study; diseases that are transmitted naturally between humans and animals including vector-borne diseases, enteric diseases and zoonotic disease of animal-origin but primarily circulating between humans, for example, SARS and H1N1). Zoonotic diseases account for varying degrees of illness in both the animal and human populations. As there are limited resources available for research, surveillance and the control and prevention of zoonotic diseases, it is necessary to prioritize diseases in order to direct resources into those with the greatest needs. But how do you define or measure greatest needs and how do you compare these needs when zoonotic diseases vary greatly in their occurrence and in their health impact on the animal and human populations? One way to do this is to identify measurable characteristics of a zoonotic disease that can be used to quantify and prioritize their importance. What I am interested in today is to get a sense of what people in the [general public / veterinary community and/or medical community] consider important characteristics of a zoonotic disease that should be considered in disease prioritization. Prioritization will no doubt differ between individuals, professionals and institutions, and this is why you have been invited here today, because I would like to understand the views of people [in the public/with your professional background] to help me formulate my final survey instrument.”*

**Focus Group Structure** – *“The first half of this focus group session will centre around a group discussion on identifying measurable characteristics of a zoonotic disease that can be used for their prioritization. In the second half of this focus group session, you will be asked to score the characteristics identified by the group in order of importance. The goal for today’s session is to come up with a list, in order of importance, of measurable characteristics that can be used to prioritize zoonotic diseases against one another.”*

*“Does anyone have any questions for me at this stage?”*

1. **Informed Consent & Demographic Data (10 minutes)**

*“Before I begin, I need to go through an informed consent procedure with you. This procedure is to help you understand the risks and benefits of participating in this study so that you can decide whether or not you wish to continue to take part. In front of you is a consent form with some of the information about the study that I have just outlined. Some of the things I would like to highlight are your rights in participating in this study:*

- *Participation is voluntary*
- *You may withdraw from this study at any time without penalty*
- *Your participation or withdrawal will not disadvantage you in any way*
- *Any information you give will be stored and used anonymously*

*Some of the risks and benefits involved in participating in this study:*

- *Risks: This study consists of a roundtable discussion and there are no known harms associated with participating in the focus group discussion, however, participation may involve discussion or questions that may make you feel anxious or uncomfortable. You have the right to refrain from answering questions or contribute to certain discussion topics that make you feel uncomfortable*
- *Benefits: You will be provided with a free meal; please feel free to eat during the session and while I am talking. While this study may not benefit you directly, the knowledge gained from this study will benefit others*

*I also want to point out the session today will be audiotaped for post-analysis, however you will not be identified at any stage of the research and all recorded and written information from today’s session will remain confidential and anonymous, meaning the data you provide me with today cannot be traced back to you individually. After you sign your informed consent forms, I will be asking you to fill in the ballot sheet in front of you, this is so that I can get an idea of the group’s demographics. These include age, gender, educational and professional background, however, I am not asking you to include any identifiable information about yourself so that your responses cannot be traced back to you. The written and recorded information you provide me with today will only be used for the purpose of the study. Is everyone comfortable with being audiotaped for today’s session and are you comfortable with providing de-identified demographic information about yourself?*

*I am going to give everyone five minutes to read through the informed consent form carefully. If you have any difficulty reading or understanding the form, please let me know and I will walk you through it. If you are willing to continue to take part in this focus group, please print your name and sign the consent form on the last page and hand it back to me. The remainder of the form is for you to keep, it has my contact details on it should you need to contact me at any time after today’s session. Once you are done, I’ll ask you to complete your demographic information forms and place it in the ballot box.”*

**3. Ground Rules (5 minutes)**

*“I am just briefly going to go through some ground rules for creating a healthy group environment for discussion, these are:*

- *Confidentiality will be maintained during the session at all times (if possible)*
- *Participate as much as possible*
- *Ask questions as they come up*
- *Turn cell phones and pagers off*
- *Respect each other’s opinions*
- *Do not interrupt speakers, allow others to finish speaking before you begin*

*Is everyone comfortable with this set of rules?”*

**4. Part 1 (Formulating a list of characteristics) (30 to 40 minutes)**

“*I am going to start off this part of the session by presenting you with two very different zoonotic diseases and the impact they have on animal and human health.”*

[Present participants with the following figure as a prompt and allowed a few minutes to read at their own pace]


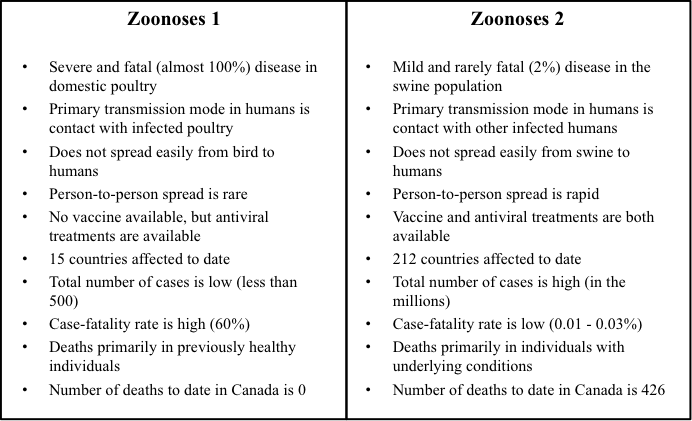


*“So you have been presented with two zoonotic diseases causing a different degree of illness in both the animal and human populations. The goal of this first part of the focus group is to identify which characteristics of zoonotic* *diseases in general, using the two diseases given as an example to start the discussion, you would consider important in disease prioritization? Please write down on a sheet of paper some of your ideas and I will ask you to share this with the group in a moment. Please do not discuss your ideas at this stage with others.* *Feel free to think outside the box and list characteristics that are not presented in this example.”*

Invite participants to share the characteristics they have identified one at a time (one characteristic per participant) and ask them why this particular characteristic has been identified. Write these down on a flip chart using the words spoken by the participant. There is no debate on items at this stage and participants are encouraged to continue to write down new ideas during the discussion to share. No characteristics identified should be eliminated. This round robin process continues until all ideas have been listed.

When the group can identify no further characteristics, condense and merge similar characteristics together. Encourage a group discussion to clarify and evaluate the characteristics identified by the group. Summarize the final list of characteristics and ask participants to rank in the second half of the focus group session.

**5. Part 2 (Rating the list of characteristics) (20 to 30 minutes)**

*“Now that we have come up with a list of potential characteristics for which to prioritize zoonootic diseases, I want you to assign a score, on a scale of 1 for least important to 9 for most important, for each characteristic (Round 1). You can rate all the characteristics as equally important (score of 9) or as equally not important (score of 1), that is, there is no limit on how many high or low scores you can give to your list of characteristics. I will give you five to ten minutes to complete this exercise and then I will ask you to read out your scores to the group when you are done. Once all the scores have been tabulated, we will then have a group discussion on the group results. In light of the group discussion, you will be asked to re-rank your responses again (Round 2).”*

[Note: only one round of rating for the professional groups due to time restriction]

**6. Closing Remarks (5 minutes)**

*“I want to thank everyone for their participation today. Are there any questions or comments?”*

*“If you don’t have any questions or comments for me now but think of some after you leave today, please feel free to contact me at any time about this study. My contact details are on the first page of the consent form. Thank you again for participating in this focus group session today.”*
